# Assessment of antifungal efficacy of itraconazole loaded aspasomal cream: comparative clinical study

**DOI:** 10.1080/10717544.2022.2067601

**Published:** 2022-05-04

**Authors:** Caroline Lamie, Enas Elmowafy, Maha H. Ragaie, Dalia A. Attia, Nahed D. Mortada

**Affiliations:** a Pharmaceutics and Pharmaceutical Technology, The British University in Egypt, Cairo, Egypt; b Pharmaceutics and Industrial Pharmacy Department, Faculty of Pharmacy, Ain Shams University, Cairo, Egypt; c Department of Dermatology, STD’s and Andrology, Faculty of Medicine, Minia University, Al Minya, Egypt

**Keywords:** Itraconazole, clinical study, candidiasis, tinea, aspasomes, cream

## Abstract

Topical conveyance of antifungal agents like itraconazole ITZ has been giving good grounds for expecting felicitous antifungal medicines. The defiance of topical delivery of this poorly water soluble and high-molecular-weight drug, however, mightily entail an adequate vehiculation. ITZ aspasomes, newer antioxidant generation of liposomes, have been designed and enclosed in a cream to ameliorate skin deposition. The proposed creams containing non-formulated ITZ or encapsulated in aspasomes (0.1% or 0.5%) were topically applied in patients with diagnosed diaper dermatitis complicated by candidiasis, tinea corporis (TC), and tinea versicolor (TVC). Placebos (void aspasomal cream and cream base) were also utilized. The obtained results for diaper rash revealed that aspasomal cream (0.5% ITZ) was eminent with respect to complete cure and negative candida culture after 10-day therapy relative to counterparts containing 0.1% ITZ aspasomes or non-formulated ITZ (0.1% and 0.5%). For tinea, the same trend was manifested in terms of ‘cleared’ clinical response in 90% of patients and absence of fungal elements after 4-week treatment. Relative to non-formulated ITZ, ITZ aspasomal cream was endorsed to be auspicious especially when ITZ concentration was lowered to half commercially available cream concentration (1%), pushing further exploitation in other dermal fungal infections.

## 1. Introduction

Globally, skin ailments affecting humans are very prevalent, prompting perplexity if not improperly remediated. In particular, outspread and recalcitrant fungal infections are critical healthcare issues as skin fungal infections can lead to life-threatening systemic infections (Nucci & Marr, 2005; Chen & Sorrell, 2007). The increment of immunocompromised patients (cancer and AIDS) results in a substantial boost in the cutaneous fungal infection incidence. There are an assortment of fungal infections affecting different populations such as candidiasis and tinea.

Candidiasis is caused by *Candida* spp, especially *Candida albicans* (CA) which is able to conquer the stratum corneum and reach the deeper dermal strata causing cutaneous candidiasis (Permana et al., 2020). Diaper dermatitis (DD) triggered by candidiasis is a frequent issue in diaper-wearing newborns (Spraker et al., 2006). Another example is superficial tinea infections that are caused by dermatophytes that affect many regions of the body such as tinea versicolor (TVC) and tinea corporis (TC). TVC is depicted by the appearance of macules, either hypopigmented or hyperpigmented, on the face, arms, trunk, and shoulders (Mendez-Tovar, 2010; Ngatu et al., 2011), while TC is a common skin infection affecting the trunk, neck, arms and legs of predisposed individuals (especially children and immunocompromised populations) (Aly & Berger, 1996).

Efficient curing of lesional skin with fungi entails exploitation of competent antifungal drugs capable of averting the rising fungal resistance, toxicity, and adverse effects. Among them, itraconazole is a wide-spectrum antifungal agent that is derived from azoles. Besides, its main metabolite; hydroxy-itraconazole has been reported to show worthwhile antifungal potential (Willems et al., 2001). Its mode of action includes the impediment of the synthesis of ergosterol; the substantial constituent of fungal cell membrane via considerable suppression of the monooxygenase in fungal cells; natural (14α demethylase, CYP51). Moreover, opposition of ITZ to lanosterol and linking of N in azole groups with the iron of heme of CYP51 are also evidenced (Ling et al., 2016; Doğan et al., 2017).

ITZ offers felicitous options against superficial and cutaneous fungal infections including resistant types and is feasible currently in 200 and 400 mg/day doses in the form of capsules and cyclodextrin solution for oral administration (Martin, 1999; Permana et al., 2021). Despite being well tolerated, some unsatisfactory side effects might occur such as constipation, headache, liver damage, and cardiac disorders when administrated orally (Kumar & Goindi, 2021). Several clinical studies conducted on ITZ have been based on its oral administration in concentrations ranged between 50 and 400 mg daily (Degreef et al., 1987; Nuijten & Schuller, 1987; Van Hecke & Van Cutsem, 1988; Bourlond et al., 1989). Yet, predominantly incompetent clinical restraint might be disclosed due to ITZ unsteady pharmacokinetics (Sardana et al., 2020; Khurana et al., 2021) in terms of erratic dose-dependent absorption (Kumar & Goindi, 2014). This could be ascribed to its high molecular weight (705.64 g/mol), low aqueous solubility and high lipophilicity (Log*P* = 5.66).

Considering these shortcomings, adverse effects and urgent need for local-targeting, topical application of ITZ seems attractive and is highly recommended. However, it is a big challenge to develop a topical ITZ platform capable of penetrating the keratinized layer of the skin (stratum corneum) and exerting its action using low concentration of drug whenever possible. Accordingly, formulating ITZ in appropriate topical nano-sized delivery systems is imperatively demanded. Referring to recent literature, some studies designed dermally oriented ITZ loaded nanocarriers (Passos et al., 2020; Kumar & Goindi, 2021; Qumber et al., 2021; Subedi et al., 2021). Despite their encouraging results, none of these works support clinically their findings.

Nanoplatforms have been extensively utilized as a treatment modality for fungal infections, attesting striking therapeutic outcomes (Bseiso et al., 2015). In the topical delivery domain, the benefits of such nanostructures are the lack of systemic side effects, achievement of high local therapeutic concentrations, enhancement of skin permeation, and improvement of drug bioavailability (Verma & Utreja, 2019; Zhang et al., 2020). Aiming at prolonging the therapeutic leverage at the area of infection, incorporating these nanocarriers in gels and creams has been demonstrated in various studies (Korting et al., 1997; Kumar & Goindi, 2021). It is worth noting that the use of topical conventional creams and gels alone has been associated with poor penetrability, inconsistent skin drug levels and hence, indigent dermal bioavailability and diminished effectiveness in terms of disease relapse or treatment failure (Verma & Utreja, 2019).

Along these lines, the design of nanovesicles, so called aspasomes, were attempted in this study to enclose ITZ for effective local delivery. Aspasomes are nano vesicular antioxidant systems aspasomes containing ascorbyl palmitate (AP) that has the ability to form stable vesicular bilayers. AP is a hydrophobic derivative of ascorbic acid, possessing the ability to reserve the antioxidant power of ascorbic acid (Moribe et al., 2011; Zariwala et al., 2014). Besides, AP assists skin penetration as a lipophilic agent (Aboul-Einien et al., 2020; Shinde et al., 2022) showing stronger skin penetration properties than ascorbic acid (Ghosh et al., 2018). For ease of application and prolongation of aspasomes, retention for better topical convenience, ITZ loaded aspasomes were incorporated in a cream base (aspasomal cream) and evaluated for the first time as a new delivery platform. To the best of our knowledge, it is barely to find a marketed topical ITZ. Even those marketed products were claimed to be illegally manufactured and promoted for ‘over the internet’ sale in some countries (Gopinath et al., 2004; Bishnoi et al., 2018).

Herein, the antifungal potential against DD complicated by candidiasis, TC, and TVC was evaluated clinically. Direct assessment of patients suffering from these fungal infections was performed, reporting response, tolerability, and apparent side effects. As the marketed ITZ cream (1%) was not available in Egypt at the time the clinical investigation was conducted, we incorporated ITZ in the prepared cream base at the same concentrations as the proposed aspasomal cream for the sake of comparison. The proposed aspasomal cream was compared with cream enclosing non-formulated ITZ, placebo (plain aspasomal cream) and control (cream base) formulae. More importantly, the impact of attempting two ITZ concentrations (0.1 and 0.5%), being lower than that utilized in the market (1%) was demonstrated.

## 2. Experimental

### 2.1. Materials

Itraconazole powder was purchased from Orkila, Spansules (Hyderabad, India). Ascorbyl palmitate was purchased from DSM (Nutritional Product GmbH, Kaiseraugst, Switzerland). Epikuron^®^ TM 200 was kindly obtained by Cargill (Minneapolis, MN). Cholesterol (CH), HPLC grade solvents (chloroform and ethanol), sodium chloride, potassium dihydrogen phosphate, disodium hydrogen phosphate, potassium hydroxide (KOH), and methyl paraben were purchased from Fisher Scientific (Waltham, MA), Acros Organics (Geel, Belgium). Glycerin was obtained from El-Nasr Pharmaceutical Company (Khanka, Egypt). Propylene glycol PP was kindly gifted from LUNA Cosmetics (Edmonton, Canada).

### 2.2. Pharmaceutical formulation

ITZ aspasomes were prepared by thin film hydration method as previously described by Lamie et al. (2022). The optimum formula consisted of AP, CH, and phospholipid in a ratio of approximately 45:45:10 w/w respectively according to our recently accepted work (Lamie et al., 2022). The optimization was performed via availing features of experimental design; Box Behnken design (Design Expert, version 12.00; Stat-Ease Inc., Minneapolis, MN). To achieve this aim, based on our preliminary study, and recently accepted work, two formulations (total amount of aspasomal components and AP percentage) and one processing (sonication amplitude) variables were investigated and the characteristics of the prepared vesicle were evaluated *in vitro*. ITZ aspasomes were characterized with respect to particle size, polydispersity index, entrapment efficiency, and morphology. An O/W cream has been chosen as a final skin product in order to prolong the retention of the aspasomes for better topical convenience. For the preparation of cream base, the conventional fusion method was used for emulsification of aqueous (KOH, glycerin, and propylene glycol) and oily (stearic acid) constituents. Methyl paraben was added at concentration of 0.01% as a preservative. The lyophilized aspasomes was gently dispersed in the cream base after being cooled down (enclosing either 0.1% or 0.5% w/w ITZ). The prepared creams were evaluated for pH, drug content, phase separation, whatever the applied centrifuge speed, spreadability, extrudability occlusive properties, and viscosity (El-Gizawy et al., 2020; Mohamed et al., 2020). The extent of penetration of the fluorescently labeled aspasomal cream in the skin strata was visualized using confocal microscopy and compared with fluorescently labeled conventional cream as previously stated in our recently accepted paper. The stability study for the prepared aspasomal creams was also performed after one year to evaluate its shelf life.

### 2.3. Clinical study

#### 2.3.1. Settings

The present study was conducted at the dermatology outpatient clinic of Minia University Hospital. One hundred and twenty patients were chosen to perform this study. Each patient signed an informed written consent before participation in the current study. The study performed on 12 separate randomized parallel double blinded major diagnostic groups and every group included 10 patients receiving different formulations in two different types of skin fungal infection (CA and tinea). ITZ was incorporated in aspasomal cream in two different concentrations (0.1%, 0.5% w/w). Protocols of treatment administration to different patients’ groups are listed in Table 1. FC1 and FC1′ contain 0.1%, 0.5% of non-formulated ITZ cream, respectively. FC2 and FC2′ contain 0.1%, 0.5% of optimized ITZ aspasomal cream, respectively. FC3 and FC4 contain optimized plain aspasomal cream and cream base respectively as controls.

**Table 1.** Protocols of treatment administration to different patients’ groups.

Treatment	Group no.	Administration form	ITZ concentration
FC1	1	Non-formulated ITZ cream	0.1%
FC1′	2	Non-formulated ITZ cream	0.5%
FC2	3	ITZ aspasomal cream	0.1%
FC2′	4	ITZ aspasomal cream	0.5%
FC3	5	Plain aspasomal cream	0%
FC4	6	Cream base	0%

#### 2.3.2. Ethical approval

The ethical committee for experimental, clinical, chemical studies (Faculty of Pharmacy, The British University in Egypt, Cairo, Egypt) approved the study with a code number: EX-2105, and the study was complied with the ethical precepts delineated in Helsinki Declaration.

#### 2.3.3. Candida albicans infection

The present study was conducted on 60 infants of both genders complaining from DD and their age ranged from 5 to 25 months. The dermatitis is depicted by the manifestation of acute erythematous patches encompassing the groin creases and areas around the anus and associated with satellite lesions (papules and pustules), pointing out napkin candidiasis. One member of the research team evaluated all of the newborns. All of the babies had their medical histories taken, which included prior candidal infections, the use of antibiotics or steroids in the past or present, recent diarrhea, and diapering practices. Inclusion criteria involved infants with DD complicated with candidiasis who did not receive any treatment prior to the study. Exclusion criteria included infants treated with steroids or antibiotics prior to the study. The severity of the diaper rash was scored by the use of three criteria including the extent, redness, and pustules as shown in Table 2 (Rebora et al., 1973).

**Table 2.** Clinical assessment of diaper rash severity.

Parameter	Scoring system/evaluation
Extent (the baby in the frog-leg posture)	Measuring the length (maximal diameter in the axis parallel to the spinal cord; cm) and the width (maximal diameter in the axis perpendicular to the length; cm)
Redness	Redness qualitative grading
	0: none
	1: mild
	2: marked
Pustules	Pustules counting
	1: one to five pustules
	2: 5–20 pustules
	3: numerous pustules
	4: confluent pustules
	5: bullae or erosions

##### 2.3.3.1. Infants’ management

All parents were advised to care about diaper rash with respect to more frequent changing of the diapers, prompt removal of dirty or wet diapers and gentle washing of the buttocks after each change. Each patient received a container of the used formula, and the caretakers were guided to apply the formula with the fingertips to the involved areas at diaper changes two times daily for a total of 10 days. After the designated treatment period, the patients were subjected to clinical examination and dermoscopic reassessments and skin scrapings.

##### 2.3.3.2. Dermoscopic and mycological examinations

It was done for all the infants at the baseline and after 10-day treatment. The diagnosis of candidal napkin dermatitis was carried out on patients' skin scrapings obtained from the affected areas. Skin scrapings were put on a glass slide, and few drops of 20% KOH were added. A slight heating was applied to the KOH preparation in order to facilitate the breakdown of the cutaneous squamous cells. The prepared slides were then inspected microscopically (light microscope; Accu-Scope #3025 five headed (A3025-5) with a digital camera; E-330 SLR, Olympus, Tokyo, Japan) (Ruocco et al., 2011). Candida presented the appearance of oval buds clusters with septate mycelium (true and pseudo).

#### 2.3.4. Tinea corporis and tinea versicolor infections

Sixty patients of both genders suffering from both TC and TVC fungal lesions were involved in this clinical study (12–22 years old). The patients who had skin lesions and did not receive topical or systemic medications within one month of enrollment were entitled for inclusion while those having skin lesions other than TC and TVC (e.g. tinea capitis or onychomycosis), and chronic illnesses (e.g. uncontrolled diabetes) were excluded. Besides, pregnant or breast-feeding females were not admitted to the clinical study. A comprehensive history was taken for all patients as follows: (1) personal history (age, gender, and employment), (2) present history (the presence of fungal infections ‘onset, course, and duration’), (3) past history (antifungal medication(s) taken previously), and (4) family history (fungal infections). The patients were clinically examined and photographed before treatment and after 4-week treatment or complete cure.

##### 2.3.4.1. Patients management

Patients were allocated into six groups; each group included 10 patients, received the used formula, and guided to apply it twice daily for 4 weeks or complete cure (Table 1). Any concurrent antifungal medication, either topical or systemic, other than the trial formulations, systemic antihistamine and corticosteroids were not permitted. The outcome of the used treatment was measured by three parameters, namely clinical cure, dermoscopic cure, and mycological cure (McNeely & Spencer, 1998; Jerajani et al., 2000). During the trial, considering safety evaluation, patients were asked to report any formula-related signs of discomfort or irritation.

##### 2.3.4.2. Clinical cure

Clinical evaluation involves the scoring of erythema; pruritus and scaling were evaluated and at baseline and after 4 weeks according to the global evaluation response listed in Table 3.

**Table 3.** Assessment of clinical cure and global evaluation response at baseline and after 4 weeks of treatment.

Scoring system	Indication
1	Cleared
2	Excellent improvement in baseline clinical signs and symptoms (90–99% cure)
3	Good improvement in baseline clinical signs and symptoms (50–89% cure)
4	Moderate (fair) improvement in baseline clinical signs and symptoms (25–49% cure)
5	Poor improvement in baseline clinical signs and symptoms (<25% cure)
6	Worse (clinical symptoms have been worsened from the baseline)

##### 2.3.4.3. Dermoscopic examination

It was done for all the patients before treatment and after 4 weeks of treatment or complete cure using dermoscope (HEINE DELTA^®^ 20 plus, Herrsching, Germany). For mycological diagnosis, skin scrapings were obtained from the affected lesions and examined as aforementioned.

A two-point scale: 0 and 1 was utilized for efficacy evaluation. The scale 0 indicates the absence of dermoscopic criteria, while the scale 1 refers to the presence of dermoscopic criteria. Referring to literature, the dermoscopic findings of TC cases depicted follicular micropustules, areas of intense erythema, and brown spots with a white-yellowish halo envelop. In addition, broken hair, wavy hair, and rarely morse code hair were revealed (Verma, 2007). The dermoscopic findings of the cases of TVC included scaling patterns (patchy, diffuse, peripheral, perifollicular, and scaling in the furrows), pigmentation patterns (nonuniform pigmentation and perilesional hyperpigmentation), and border patterns (clearly demarcated border), with in conspicuous ridges and furrows (Gupta et al., 2003).

##### 2.3.4.4. Mycological examination

A light microscope was used to perform a direct mycological investigation after 28 days. The absence of fungal components in the skin scrapings is indicative of the mycological cure. The efficacy was assessed on a two-point scale; 0 and 1. The scale 0 indicates the lack of fungal elements and the scale 1 indicates the presence of fungal elements.

### 2.4. Statistical analysis

A one-way ANOVA test was performed to compare the six groups, followed by a post hoc statistical analysis between each pair of groups. Paired samples *t*-test between before and after treatment was done in each group. Fischer’s exact test was carried out for qualitative data between groups. Wilcoxon’s signed rank test between before and after treatment was also conducted in each group. Significant level was set at *p* value <.05.

## 3. Results

**Figure 1.**
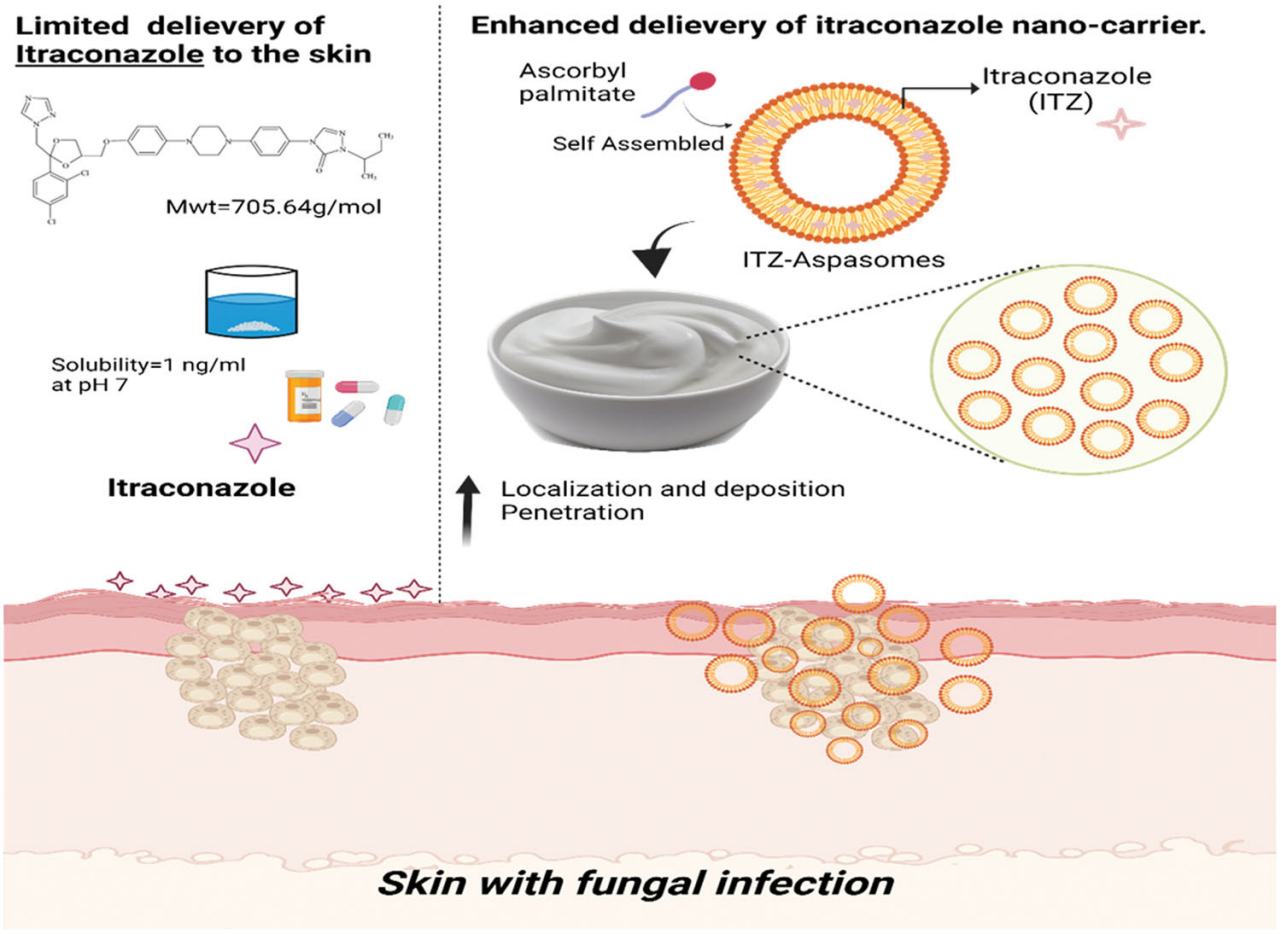
Schematic diagram representing the enhanced topical delivery of Itraconazole aspasomal cream.

The present clinical study was designed to investigate if the encapsulation of ITZ in lipid-based vesicles; aspasomes and their incorporation in a cream base could lower its antifungal concentration, in comparison with the same ITZ concentration in marketed cream. Incorporating the proposed aspasomes in a cream base could prolong their topical retention and deposition. Figure 1 demonstrates the schematic diagram representing the enhanced topical delivery of ITZ aspasomal cream. Along these lines, the ITZ concentration in the administered unencapsulated ITZ cream (reference product) or ITZ aspasomal cream (test product) was varied to demonstrate whether the application of ITZ in cream at lower doses than that in the marketed product would attain a comparable consequence. Besides, patients in the study would be given two placebo formulae (plain aspasomal cream and cream base) in order to ascertain the adequate sensitivity of the clinical study to differences between treatments. The mycological evaluation was performed on subjects with two types of fungal infections (candidiasis and tinea).

### 3.1. Pharmaceutical formulation

**Figure 2.**
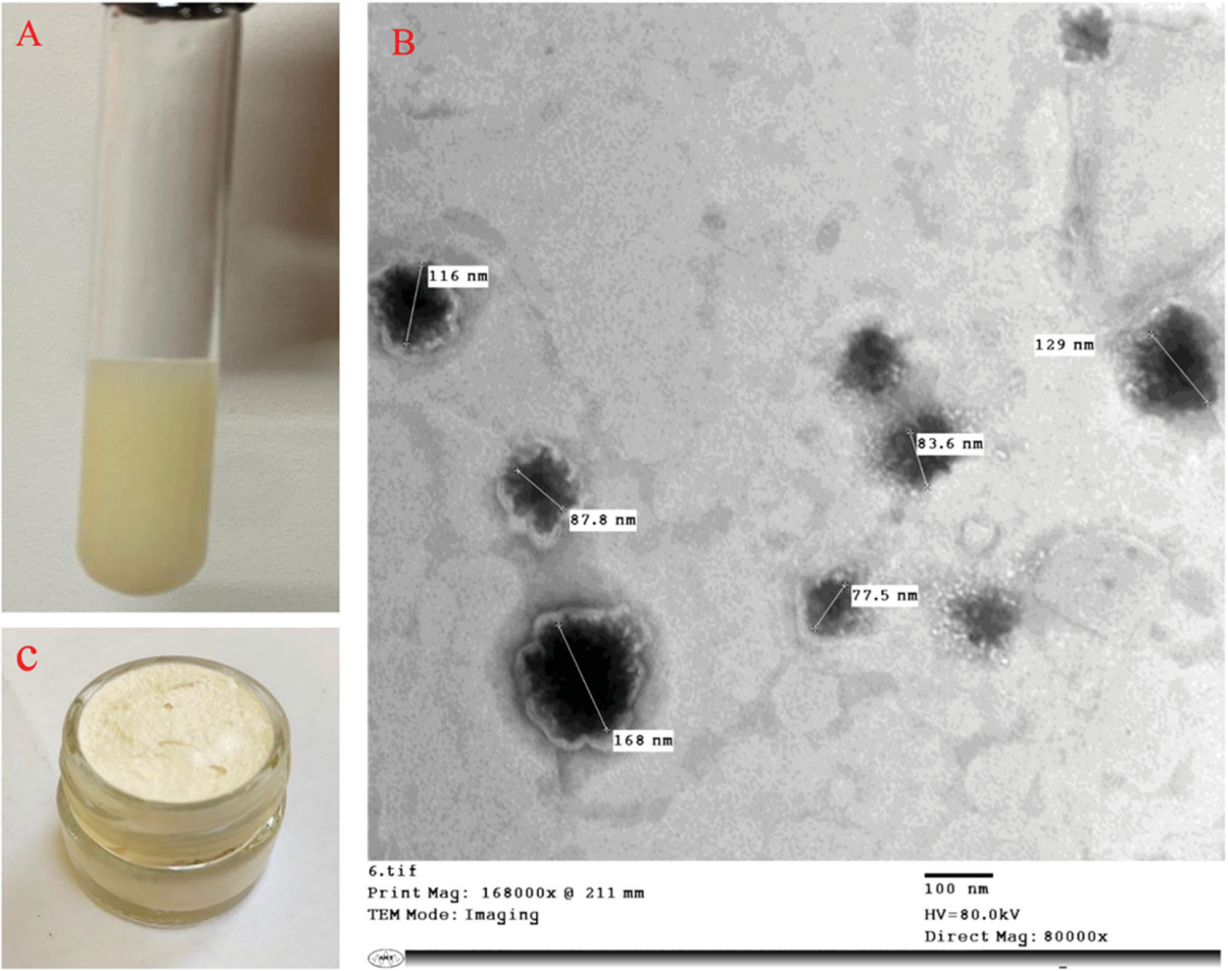
Illustration of itraconazole: (A) itraconazole aspasomes, (B) transmission electron micrograph of itraconazole aspasomes, and (C) itraconazole aspasomal cream.

ITZ was felicitously encapsulated in the optimized aspasomes, with a nano size ˂200 nm, a negative surface charge and high entrapment potential exceeding 90%. As revealed in TEM, the aspasomes appeared as a spherical and smooth vesicle (Figure 2). As for the prepared aspasomal creams (0.1% aspasomal cream was previously prepared and evaluated in our recently accepted paper (Lamie et al., 2022) and 0.5% aspasomal cream), both systems were found to be smooth, and non-gritty and capable of incorporating >95% ITZ (95.12%±0.24 and 96.41%±1.1 for 0.1% and 0.5%, respectively; (*p*> .05)). Besides, both creams showed similar features with respect to pH (around 7; 6.84 ± 0.21 and 6.84 ± 0.32 for 0.1% and 0.5%, respectively), appropriate spreadability (respective values of 443.3%±6.5 and 442.4%±5.1 for 0.1 and 0.5% aspasomal creams), considerable occlusion (respective values of 78.26%±5.75 and 79.06%±3.7 for 0.1 and 0.5% aspasomal creams), excellent extrudability (respective values of 97.5%±0.82 and 96.6%±1.2), and shear-thinning viscosity (*p*> .05) (Table S1, and Figure S1). A representative confocal laser scanning microscopy (CLSM) images for skin treated with ITZ cream and aspasomal cream (Figure S2) revealed the higher skin penetrability of the fluorescently labeled aspasomal cream relative to the conventional cream.

The stability study after one year for 0.1% and 0.5% aspasomal cream also revealed that the ITZ concentration was found to be (95%±1.2) and (96.2%±0.45), respectively (*p*> .05) with good homogenous appearance with no visual color changes or phase separation. Moreover, there is no significant difference (*p*> .05) detected in pH value, spreadability, occlusive effect, and extrudability as shown in Table S2. Regarding the viscosity of the cream (0.1%, 0.5%), pseudoplastic (shear-thinning) behavior was revealed (*p*> .05).

### 3.2. *Candida albicans* infection

**Figure 3.**
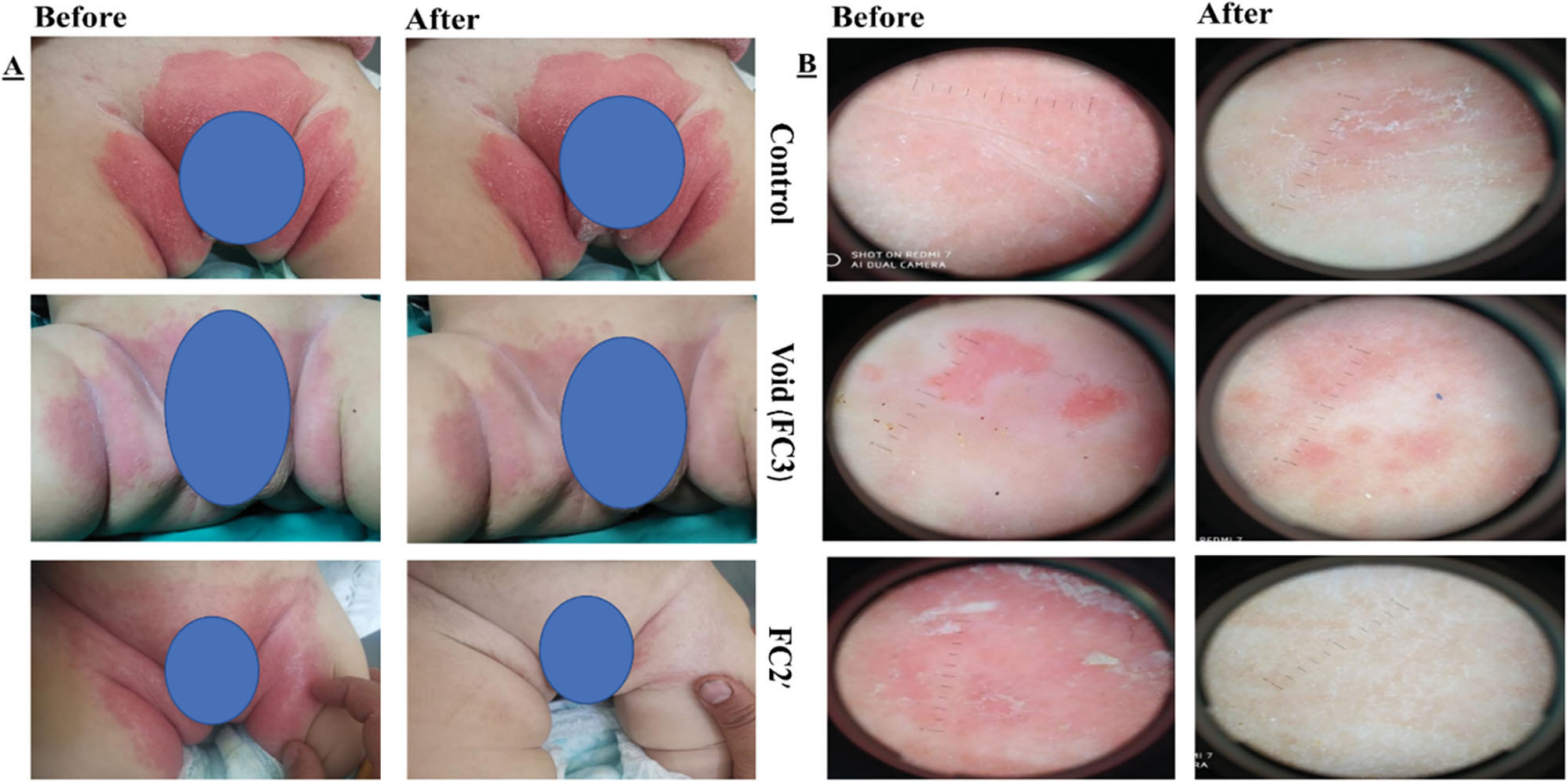
(A) Photographic photos of control, void, effective aspasomal cream before and after treatment in candidiasis. (B) Dermoscopic picture before treatment showed the presence of oval buds’ clusters with pseudo and true septate mycelium, and its disappearance after treatment indicates the efficacy of aspasomes encapsulated ITZ (FC2′).

The present study was conducted on 60 infants (six groups; 10 infants each). Concerning the initial evaluation, the dermoscopic examination showed white scales, pustules (circular pale white spots) and erythematous background in 100% of patients. Besides, skin scrapings revealed the presence of *C. albicans* in 100% of patients. The demographic data among the designated groups showed no statistical significance (*p*>.05) (Table S3), ensuring no influence on the resolution of candidal DD. The formulations were well received by the volunteers, whose parents reported no adverse effects while using them or afterwards. Clinical evaluation (area of rash, redness, and pustules) and dermoscopic and mycologic evaluations of napkin candidal dermatitis are listed in Tables 4 and 5, respectively. Representative photographic images and dermoscopic pictures of patients infected with CA before and after treatment are revealed in Figure 3.

**Table 4.** Clinical evaluation of napkin candidal dermatitis in 60 patients before and 10 days after the start of treatment.

Clinical assessment	Study formulae						
	FC1	FC1′	FC2	FC2′	FC3	FC4	*p* Value^a^
Area of rash (cm^2^)							
Before	46 ± 9.5	46.2 ± 11.9	44.4 ± 10.2	47.1 ± 8.6	46 ± 12.9	42.3 ± 10.1	.931
After	18.2 ± 6.4	26.8 ± 6.2	17.1 ± 6.8	0 ± 0	43.2 ± 12.2	42.3 ± 10.1	<.001
*p* Value^b^	<.001	<.001	<.001	<.001	.021	1	
Redness							
Before							
None	0 (0%)	0 (0%)	0 (0%)	0 (0%)	0 (0%)	0 (0%)	.997
Mild	4 (40%)	3 (30%)	3 (30%)	2 (20%)	3 (30%)	3 (30%)	
Marked	6 (60%)	7 (70%)	7 (70%)	8 (80%)	7 (70%)	7 (70%)	
After							
None	0 (0%)	0 (0%)	0 (0%)	10 (100%)	0 (0%)	0 (0%)	<.001
Mild	10 (100%)	10 (100%)	8 (80%)	0 (0%)	4 (40%)	3 (30%)	
Marked	0 (0%)	0 (0%)	2 (20%)	0 (0%)	6 (60%)	7 (70%)	
* p* Value^b^	.014	.008	.025	.003	.317	1	
Pustules							
Before							
0	0 (0%)	0 (0%)	0 (0%)	0 (0%)	0 (0%)	0 (0%)	.396
1–5	0 (0%)	2 (20%)	0 (0%)	0 (0%)	0 (0%)	0 (0%)	
5–20	0 (0%)	0 (0%)	0 (0%)	0 (0%)	0 (0%)	0 (0%)	
Innumerable	0 (0%)	1 (10%)	1 (10%)	0 (0%)	0 (0%)	0 (0%)	
Confluent	5 (50%)	3 (30%)	4 (40%)	2 (20%)	5 (50%)	4 (40%)	
Bullae/erosion	3 (30%)	4 (40%)	5 (50%)	8 (80%)	5 (50%)	6 (60%)	
After							
0	0 (0%)	2 (20%)	0 (0%)	10 (100%)	0 (0%)	0 (0%)	<.001
1–5	0 (0%)	0 (0%)	0 (0%)	0 (0%)	0 (0%)	0 (0%)	
5–20	4 (40%)	3 (30%)	2 (20%)	0 (0%)	0 (0%)	0 (0%)	
Innumerable	6 (60%)	4 (40%)	7 (70%)	0 (0%)	0 (0%)	0 (0%)	
Confluent	0 (0%)	1 (10%)	1 (10%)	0 (0%)	4 (40%)	4 (40%)	
Bullae/erosion	0 (0%)	0 (0%)	0 (0%)	0 (0%)	6 (60%)	6 (60%)	
* p* Value^b^	.007	.004	.004	.003	.317	1	

Fisher’s exact test for qualitative data between groups. Wilcoxon’s signed rank test between before and after treatment in each group. Significant level at *p* value ≤.05.

^a^ One-way ANOVA test between the six groups followed by post hoc analysis between each two groups.

^b^ Paired samples *t*-test between before and after treatment in each group.

**Table 5.** Dermoscopic and mycologic evaluations of napkin candidal dermatitis in 10 patients/group before and 10 days after the start of treatment.

	FC1	FC1′	FC2	FC2′	FC3	FC4
Dermoscopic scoring						
Before	10/10	10/10	10/10	10/10	10/10	10/10
After	10/10	10/10	10/10	0/10	10/10	10/10
Skin scrapings						
Before	10/10	10/10	10/10	10/10	10/10	10/10
After	10/10	10/10	10/10	0/10	10/10	10/10

Clinical evaluation (Table 4) revealed that 100% of the infants in group receiving FC2′ formula (ITZ aspasomal cream) showed a promising amelioration in all clinical parameters of napkin candidiasis.

Regarding area of rash, groups treated with ITZ in non-formulated and encapsulated forms in creams (FC1, FC1′, FC2, and FC2′; groups 1–4) showed an extremely statistical difference (*p*<.001). It is also noticed that group treated with void cream containing plain aspasomes (FC4) showed a significant decrease in the area of rash (*p*<.05), pinpointing the prominence of the presence of AP in aspasomes, in exerting an antioxidant potential and hence healing promotion. Similarly, the percentage of cure from redness and pustules among groups treated with ITZ encapsulated aspasomal formulation and the non-formulated 0.5% ITZ revealed a highly significant difference (*p*<.05). Interestingly, the absence of redness or pustules was noticed in 100% of patients receiving high concentration of ITZ encapsulated in aspasomal cream (0.5%; FC2′). There are notable improvements (*p*<.05) in therapy for fungal candidiasis after 10 days of treatment while increasing the concentration of ITZ from 0.1% (FC1′) to 0.5% (FC2′) in the aspasomal cream. In contrast, other groups (FC1, FC1′, FC2, FC3, and FC4) failed to present any signs of betterment in all clinical criteria at 10-day therapy in 100% of the infants.

Further inspection of Table 4 revealed that the percentages of cured infants throughout the study were significantly higher (*p*<.05) in groups 3 and 4 (FC2 and FC2′) than in groups 1, 2, 5, and 6 (FC1, FC1′, FC3, and FC4), highlighting superiority of ITZ aspasomal cream over ITZ cream and plain formulae. Considering the leverage of ITZ concentration, the percentage of cured cases from the start to the completion of the clinical study was significantly greater in group 4 (FC2′) relative to group 3 (FC2) (*p*<.05). The application of higher concentration of ITZ (0.5%) appeared to present complete cure.

For dermoscopic and mycologic evaluation (Table 5), culture specimens tested negative for *C. albicans* after 10-day therapy in 100% of infants in group 4 receiving FC2′ formula. However, dermoscopic evaluation and skin scrapings remained positive for *C. albicans* after 10 days of therapy in 100% of infants in other groups (FC1, FC1′, FC2, FC3, and FC4).

### 3.3. Tinea corporis and tinea versicolor infection

**Figure 4.**
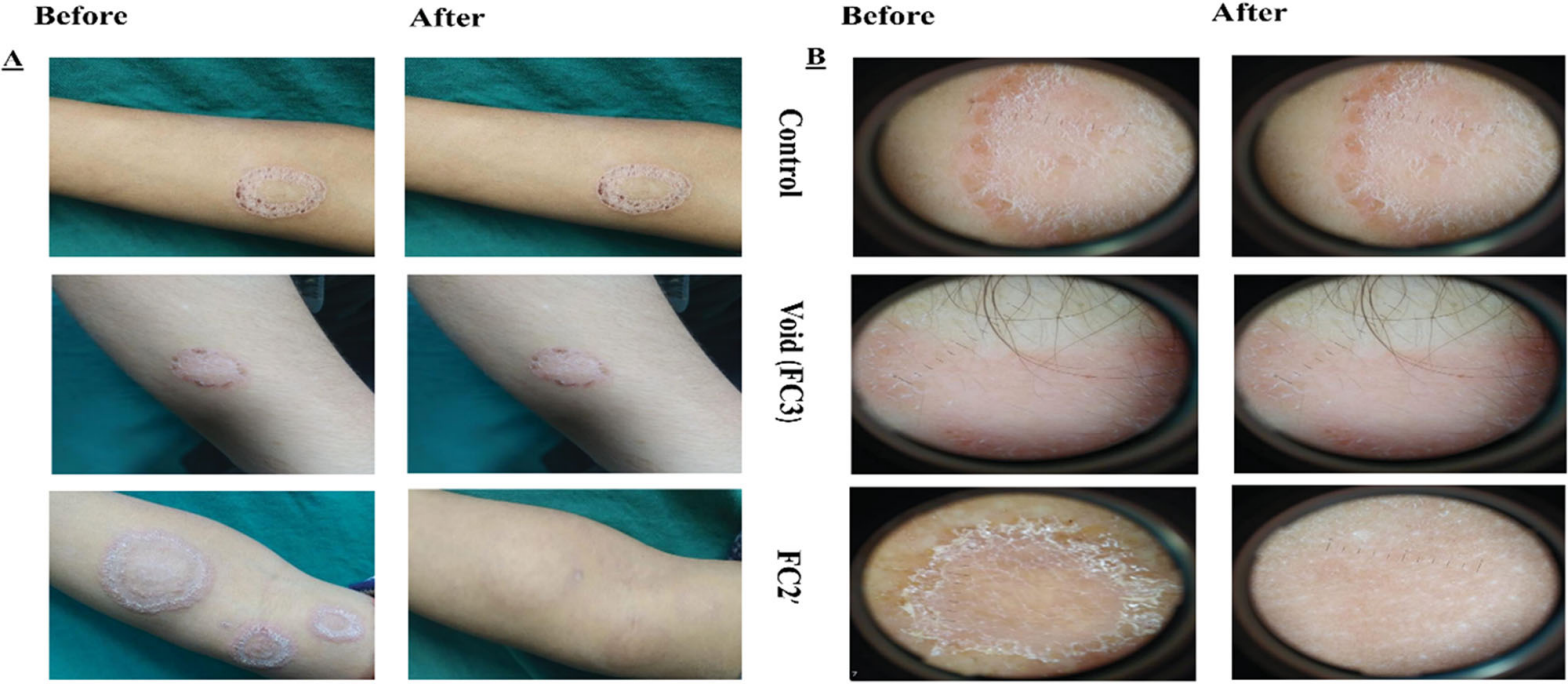
(A) Photographic photos of control, void, effective aspasomal cream (FC2′) before and after treatment in TC. (B) Dermoscopic picture before treatment showed the presence of circinate erythematous scaly lesions with elevated raised border TC, and its disappearance after treatment indicates the efficacy of aspasomes encapsulated ITZ cream FC2′.

**Figure 5.**
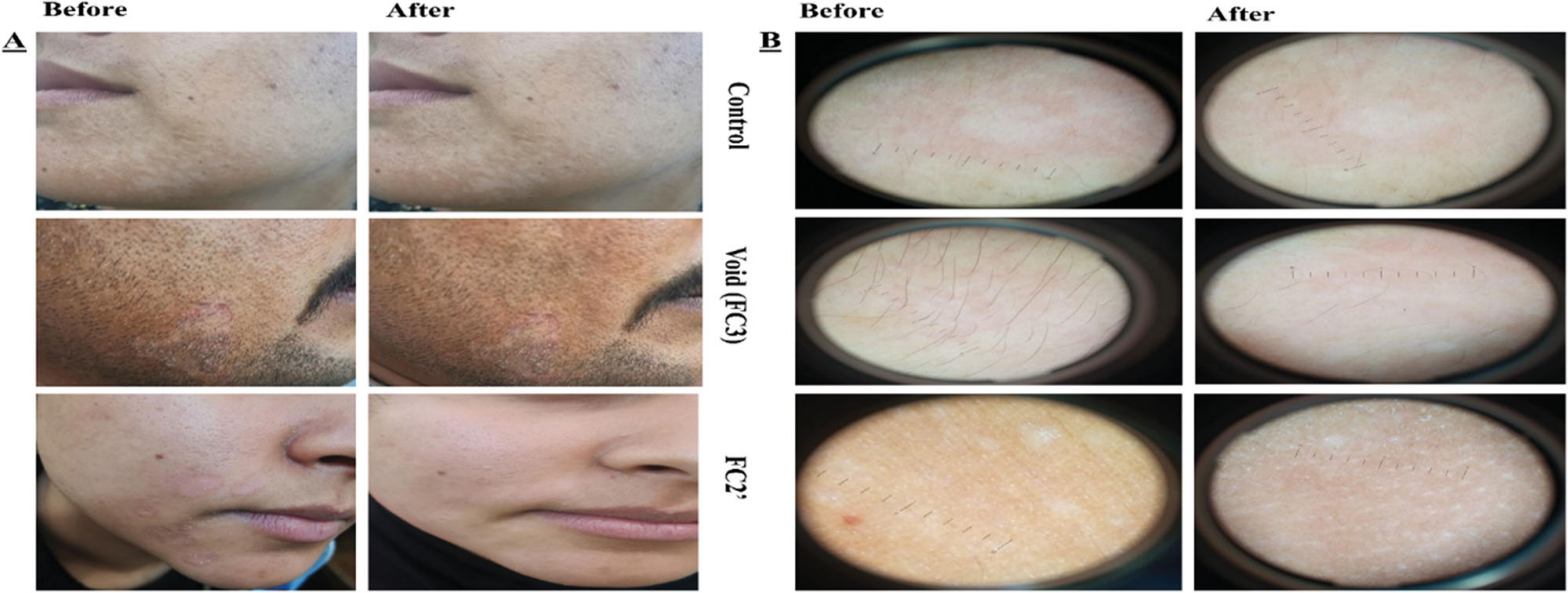
(A) Photographic photos of control, void, effective aspasomal cream (FC2′) before and after treatment in TVC. (B) Dermoscopic picture showed the presence of appeared clinically as hyperpigmented or hypopigmented, round to oval lesions TVC, and its disappearance after treatment indicates the efficacy of aspasomes encapsulated ITZ cream FC2.

Demographic data of the 60 patients included in the study are described in Table S4. No significant differences in demographic data were found among the six groups (*p*>.05). Thirty patients were diagnosed with TC which appeared as circinate lesions with erythematous, scaly and raised border on the predilection sites (face, trunk, and extremities) (Mathur et al., 2019) and the other 30 were presented with TVC manifested clinically as round to oval lesional skin of trunk, upper arms, and face (hyperpigmented or hypopigmented) (Bhat et al., 2019). The diagnosis was confirmed by positive KOH scrapings showing the fungal elements in the form of hyphae. Clinical scoring of tinea (TC and TVC) in 10 patients/group before and 4 weeks after the start of treatment and efficacy assessment (global evaluation response) are listed in Tables 6 and 7, respectively. Representative photographic images and dermoscopic pictures of patients infected with TC and TVC before and after treatment are revealed in Figures 4 and 5, respectively.

**Table 6.** Clinical Score of tinea (TC and TVC) in 10 patients/group before and 4 weeks after the start of treatment.

	FC1	FC1′	FC2	FC2′	FC3	FC4	*p* Value^a^
Clinical score Before							
Median	9	9	9	9	9	9	.800
IQR	(8–9)	(7–9)	(8.8–9)	(8.8–9)	(8–9)	(8.8–9)	
Clinical score After							
Median	8	7	6	0	9	9	<.001*
IQR	(7–8)	(5.8–7)	(5.8–6)	(0–0)	(8–9)	(8.8–9)	
*p* Value^b^	.002*	.003*	.002*	.003*	1	1	

* Significant level at *p* value <.05.

^a^ The Kruskal–Wallis test between the six groups followed by Mann–Whitney’s test between each two groups.

^b^ Paired samples *t*-test between before and after treatment in each group.

**Table 7.** Global evaluation response between the six groups.

	FC1	FC1′	FC2	FC2′	FC3	FC4	*p* Value
Response							
Cleared	0 (0%)	0 (0%)	0 (0%)	9 (90%)	0 (0%)	0 (0%)	<.001*
Excellent	0 (0%)	0 (0%)	0 (0%)	1 (10%)	0 (0%)	0 (0%)	
Good	0 (0%)	0 (0%)	5 (50%)	0 (0%)	0 (0%)	0 (0%)	
Fair	2 (20%)	4 (40%)	5 (50%)	0 (0%)	0 (0%)	0 (0%)	
Poor	8 (80%)	6 (60%)	0 (0%)	0 (0%)	8 (80%)	9 (90%)	
Worse	0 (0%)	0 (0%)	0 (0%)	0 (0%)	2 (20%)	1 (10%)	

Fisher’s exact test for qualitative data between groups.

*Significant level at p value <.05: .

The six groups were coincided in the sign and symptom score at the baseline ‘9’. Median sign and symptom score of tinea was ‘9’ at the baseline, that was greatly diminished reaching ‘0’ in group 4 (FC2′), whereas it became ‘8’, ‘7’, and ‘6’ for groups 1, 2, and 3 (FC1, FC1′, and FC2) at the end of the treatment, respectively. For groups 5 and 6 (FC3 and FC4) treated with placebo products (aspasomal cream and cream base), the score remained unchanged ‘9’. A statistically significant difference was obtained after treatment among all groups (*p*< .001) and there is a statistical difference between before and after treatment within the same group for 1, 2, 3, and 4 groups (FC1, FC1′, FC2, and FC2′; *p*< .05) as revealed in Table 6.

On comparison of global evaluation response among all groups (Table 7), there was a considerable clinical improvement in groups treated with ITZ formulae (FC1, FC1′, FC2, and FC2′; *p*< .001) compared to groups received placebo products (FC3 and FC4) that showed ‘poor’ and ‘worse’ evaluations. Contrasting the results of global evaluation response between groups 3 and 4 (FC2 and FC2′), receiving the encapsulated aspasomal cream with the low and high ITZ concentrations (0.1% and 0.5%), respectively, revealed that 50% of patients showed ‘good’ evaluation while the rest of patients (50%) showed ‘fair’ evaluation in group 3 with respect to 90% of patients of group 4 that showed ‘cleared’ evaluation. Only 10% of patients in group 4 showed ‘excellent’ response. It is worth noting that group 1 (FC1) receiving the low concentration of non-formulated ITZ cream (0.1%) showed 20%percentage of ‘fair’ response and 80% of ‘poor’ response. Increasing ITZ non-formulated concentration in cream (0.5%) in group 1 (FC1′) resulted in a slight improvement for the same responses (respective percentages of 40% and 60%).

As for dermoscopic evaluation, there was a significant dermoscopic improvement at the end of 4 weeks among the six groups (*p*< .001). No fungal infection was noticed at the end of the therapy in 100% of patients (*N* = 10) in group 4 (FC2′). However, for the other groups (1, 2, 3, 5, and 6; FC1, FC1′, FC2, FC3, and FC4), fungal criteria were present after 4 weeks of therapy in 100% of patients (*N* = 10), without any significant cure detected during the course of the study as shown in Table 8.

**Table 8.** Dermoscopic evaluations of TC and TVC in 10 patients/group before and 4 weeks after the start of treatment.

Dermoscopic cure	FC1	FC1′	FC2	FC2′	FC3	FC4
Before						
Score 0	0 (0%)	0 (0%)	0 (0%)	0 (0%)	0 (0%)	0 (0%)
Score 1	10 (100%)	10 (100%)	10 (100%)	10 (100%)	10 (100%)	10 (100%)
After						
Score 0	0 (0%)	0 (0%)	0 (0%)	10 (100%)	0 (0%)	0 (0%)
Score 1	10 (100%)	10 (100%)	10 (100%)	0 (0%)	10 (100%)	10 (100%)
*p* Value	1	1	1	.002*	1	1

Wilcoxon’s signed rank test between before and after treatment in each group.

* Significant level at *p* value <.05.

Concerning assessment of mycological cure (Table 9), skin scrapings became negative for fungal elements after 4 weeks of therapy in 100% of patients (*N* = 10) in group 4 (FC2′). Likewise, dermoscopic results, skin scrapings remained positive for fungal components after 4 weeks of therapy in 100% of patients (*N* = 10), without any significant cure detected at the end of the 4 weeks in the rest groups (1, 2, 3, 5, and 6; FC1, FC1′, FC2, FC3, and FC4).

**Table 9.** Mycologic evaluations of TC and TVC in 10 patients/group before and 4 weeks after the start of treatment.

Mycological cure	FC1	FC1′	FC2	FC2′	FC3	FC4
Before						
– ve	0 (0%)	0 (0%)	0 (0%)	0 (0%)	0(0%)	0 (0%)
+ ve	10 (100%)	10 (100%)	10 (100%)	10 (100%)	10(100%)	10 (100%)
After						
– ve	0 (0%)	0 (0%)	0 (0%)	10 (100%)	0 (%)	0 (0%)
+ ve	10 (100%)	10 (100%)	10 (100%)	0 (0%)	10 (100%)	10 (100%)
*p* Value	1	1	1	.002*	1	1

Wilcoxon’s signed rank test between before and after treatment in each group.

* Significant level at *p* value <.05.

## 4. Discussion

Amid the rising skin illnesses, superficial fungal infections are the most frequently encountered dermal ailments such as candidiasis and tinea. *Candida albicans* is a normal commensal yeast of human microflora which is found mainly on the surface of mucosal membranes like gastrointestinal, respiratory, and genitourinary tracts. It is a major species that are responsible for 46.3% of candidiasis infection (Pedrosa et al., 2014). Diaper dermatitis is a main reason for candidiasis infection due to direct contamination of stool containing CA with inflamed skin from diapers. The infant defense response and the how deep the inflammation affect the integrity of SC are critical factors that determine whether candida can stay in SC or reach the deeper layers (Spraker et al., 2006). TC and TVC are two types of fungal infections that affect mainly the superficial dermal strata and present in the trunk and extremities (Dadar et al., 2018). In tropical climates especially in places characterized by poverty and inadequate hygiene, TC and TVC are widespread in teenagers and young adults.

In the skin fungal infections arena, oral administration of ITZ capsules has been attempted in several clinical studies. These reports have utilized various conventional ITZ dosage regimens and standard treatment durations. For instance, Degreef et al. (1987) utilized 50–100 mg ITZ daily and stated that when the dose of ITZ increased from 50 mg to 100 mg in patients suffered from acute, chronic, and recurrent dermatophytosis, faster response and better clinical and mycological improvements had been fulfilled. Likewise, ITZ was reported to be less active against CA when given orally in 100 mg doses and the authors recommended and evidenced the better performance of oral administration of two doses of 200 mg ITZ per day for a total of 4 weeks (Drake et al., 1996). Considering the duration of treatment, Nuijten & Schuller (1987) declared that 100 mg of ITZ for two weeks was effective for lesional skin with fungi. Doubling ITZ dose was also attempted by Hay & Clayton (1987). The authors recorded the superiority of 200 mg ITZ oral doses for a period five days over 100 mg doses in the management of TVC.

As the main target site for these mycoses is the stratum corneum (Estrada, 1987), the researchers need to respond to such provocation of delivering drugs across the dermal barrier at a therapeutically relevant concentration for the management of such ailments. In light, developing topical platforms present attractive therapeutic aspects in terms of lower harm or incidence of serious adverse effects of systemically administered antifungal medications (oral or injectable), better efficacy and the ability to simply terminate the medication when necessary, hence, greater patient compliance (Estrada, 1987; Rezabek & Friedman, 1992). Indeed, effectiveness of any topically applied antifungal agent mainly depends on its concentration in the infected area, its activity spectrum and safety (Kalavathy et al., 2005; Garg et al., 2015).

In general, concerning oral dosage regimen of ITZ, high oral doses must be administered 2–3 times daily over a period 3–6 months. Such high doses over long durations inevitably result in the reported adverse reactions or toxicity. Also, when administered topically to the eye, it was applied at concentration of 1% (Ho et al., 2020). However, this is not the case in our study. Topical delivery of ITZ was attempted in this study using low doses (0.1% and 0.5%) for short durations (10 days) aiming at reducing the toxicity.

Along these lines, several types of nanocarriers have been investigated for topical delivery of ITZ. Most promising is the lipid-based nanostructures like lipid vesicles that have been capable of providing enhanced penetration and deposition within the skin layers and improved solubilization and dermal bioavailability of this lipophilic drug. Lately, utilization of the antioxidant vesicles; aspasomes have gained considerable attention. Pivotal targets to formulate this lipophilic antioxidant system are comprised of disposition of the dermal tissue and permeation enhancement for this high molecular weight drug (Kaur & Kakkar, 2010). Many outstanding features have been demonstrated for the exploitation of aspasomes for topical conveyance, such as enhancing the permeation through SC, safety, biocompatibility, minimizing the applied drug amounts, and maximizing the therapeutic competence (Amer et al., 2020; Khalil et al., 2021). AP, the main component of aspasomes, was bifunctional acting as a lipid bilayer forming agent and stabilizer and antioxidant. The implication of oxidative stress in skin fungal diseases and changes of antioxidant–oxidant balance cannot be underestimated (Hatem et al., 2018). The antioxidant potential of these vesicles could play an influential part in reducing overwhelming inflammation associated with worsened fungal infections via neutralizing the involved reactive oxygen species (Ozturk et al., 2013). So far, no previous work has elucidated the impact of aspasomes on enhancing ITZ dermal localization and combating skin infection associated with inflammation.

Compared with conventional non-formulated powder that was administered in high doses orally in the form of capsules, utilizing nanovesicles like aspasomes for ITZ topical delivery could maximize therapeutically relevant concentrations at the site of action with the possibility to reduce the applied dose. ITZ was successfully enclosed within aspasomes at >90% encapsulation efficiency that could be credited to its lipophilicity that promote its amalgamation with the lipidic AP bilayer. It is worth mentioning that particle size ˂200 nm, a negative surface charge ensured efficient topical delivery (Kaur & Kakkar, 2010).

However, the liquid nature and low viscosity of aspasomes necessitate its incorporation in a suitable platform in order to prolong its contact with the infected areas of the skin. Accordingly, aspasomal cream was developed and investigated for the first time, with the aim of prolonging the skin retention and hence boosting drug-skin availability. *In vitro* appraisal was performed for the aspasomal creams to elucidate their conformity, effectiveness, and compliance. The smooth appearance of the prepared cream and the absence of any coarse particles or phase separation highlighted the symmetrical allocation of lyophilized powder in the cream base. This was further supported by high percentages of ITZ content (around 95%). The proposed liposomal cream showed an appropriate occlusive effect. It is come in accordance with Jukanti et al. (2011), that it could be linked to the nano-formulation and formation of thin film on skin post-application that covers the pores hindering water evaporation. This yielded an efficient physical barrier that could enhance drug permeation across the skin (Rapalli et al., 2020). Excellent spreadability and extrudability were also revealed, contributing to therapeutic efficacy (Shah et al., 2021). Furthermore, the congruent pH of the cream with that of pH of skin ascertained cream tolerability and non-irritancy. Moreover, the proposed aspasomal cream followed a non-Newtonian flow, pinpointing the low resistance when applying high shear stress and ease of rubbing, an issue that is highly desirable in topical dosage forms (Al-Suwayeh et al., 2014; Matangi et al., 2014). The high viscosity of the prepared aspasomal creams ensured the appropriate consistency of the cream and the formation of uniform and consistent film on the skin. The results of stability study revealed the insignificant change in drug content and the tested parameters over the stated storage period.

For clinical appraisal of the conveyance prospect of the proposed ITZ aspasomal cream, it is topically applied on skin of patients with diagnosed DD complicated by candidiasis and TC and TVC. For the sake of comparison, conventional cream (containing non-formulated ITZ) and placebos (plain aspasomal cream and cream base) were also topically applied. As the prime solicitude of clinical assessment of topically applied of nanoplatforms is their tolerability and lack of irritancy, safety evaluation was performed. The ITZ aspasomal creams (FC2 and FC2′) were found to be competent against the tested species, performing better that conventional cream and placebos. Importantly, the patients' compliance was excellent, and no adverse effects were noted.

As for DD complicated by candidiasis, the rash area, redness, presence of pustules was clinically assessed and was found to be decreased in infants treated with creams containing both ITZ forms (non-formulated and encapsulated in aspasomes). In particular, treatment using aspasomal cream containing 0.5% ITZ resulted in plenary clinical cure (100% patients) after 10-day treatment. Interestingly, the cream containing void aspasomes showed some therapeutic consequence. In addition to carrier task that could promote the superior skin deposition of the therapeutic agent, aspasomes as an antioxidant delivery platform could be successful in neutralizing reactive oxygen species at inflammatory sites (Odeniyi et al., 2020).

Similarly, for tinea, ITZ aspasomal creams (FC2 and FC2′) were attested to be a felicitous remedy, showing complete cure for the two species under study (TC and TVC) after 4-weeks treatment despite of the topical application of lower concentrations (0.1 and 0.5%) than that of marketed product (1%). For all fungal infections under investigation, non-formulated ITZ creams (FC1 and FC1′), whatever was ITZ concentration, failed in presenting satisfactory therapeutic outcomes. This could be due to the large molecular weight of ITZ (M.wt.=705.6 g/mol) that hindered its permeation through the dermal strata. It is well-documented that high molecular weight drugs >500 Da possess poor permeation across the stratum corneum layer of the skin. Despite its high molecular weight, however, high lipophilicity of ITZ enables it to accumulate in the keratin layer and reside in skin (Ho et al., 2020). The great affinity of azole drugs including ITZ to keratin layer serving as a reservoir for drugs with negligible deposition into epidermis and dermis was stated earlier (Jukanti et al., 2011; El-Sheridy et al., 2019; Aboul-Einien et al., 2020). In our study, ITZ loaded aspasomal cream was clinically assessed using non-formulated ITZ loaded cream for the sake of comparison. The incorporation of non-formulated ITZ in cream showed therapeutic efficacy. This could be credited to the incorporation of glycerin and propylene glycol in the cream base that was reported to possess permeation enhancement effects (Nair et al., 2013; Kianvash et al., 2017). In this sense, formulating nano-sized delivery platforms enclosing ITZ can be driven directly into the deep skin strata and an amplified therapeutic potential of the aspasomal cream can be anticipated. It should be mentioned that testing *ex vivo* permeation of conventional cream and aspasomal cream was performed in our recently accepted paper (Lamie et al., 2022). ITZ in oily solution (olive oil) was also assessed for skin permeation. The results revealed that ITZ deposition in stratum corneum from different formulae was 5%, 18%, and 40%. Besides, only aspasomal cream showed 56% deposition in the epidermis whereas ITZ oily solution and conventional cream showed 0% and 14%, respectively (Lamie et al., 2022).

In sum, topical application of ITZ in the form of aspasomal cream could proffer prompt relief in infectivity, greater degree of improvement, more sustained benefit compared to cream containing non-formulated ITZ. Enhanced localization and permeation of ITZ loaded aspasomes in cream appeared to be related to the nanosized of aspasomes and their incorporation in the cream base. Indeed, utilizing cream for topical delivery was reported to increase the solubilization capacity of lipophilic drugs, and perform an occlusive effect, potentiating their permeation (Fluhr et al., 2008). Interestingly, the prepared topically applied ITZ in the form of aspasomal cream was found to provide immediate reductions in fungal infectivity with no reported systemic adverse effects. It is worth mentioning that, in our recently accepted work (Lamie et al., 2022), when ITZ aspasomal cream was tested *ex vivo* on mice skin, no detectable ITZ concentrations were found in both the dermis and receptor compartment, pinpointing its topical delivery and the lack of its diffusion into non-targeted tissues. The increased accumulation and deposition of ITZ onto the stratum corneum resulted in the formation of a drug depot for optimal permeation into the epidermis; an issue that is highly warranted for the management of relevant dermatological infections.

## 5. Conclusions

ITZ aspasomes prepared with the antioxidant bilayer forming agent; AP was successfully prepared to attain an optimum size for topical delivery; ˂200 nm, an appropriate high entrapment efficiency and spherical shape. Incorporating aspasomes in cream played a complementary effect in achieving enhanced skin localization. Decreasing the marketed product ITZ concentration (1%) to half (0.5%) in aspasomal cream yielded a better proficient clinical performance for infants with proven DD associated with candidiasis and patients with proven TC and TVC relative to the non-formulated ITZ. The results of this clinical assessment set that the topical application of ITZ nanoplatform would be recommended as a prosperous and safe therapy for fungal infections caused by other species.

## Authors contributions

Caroline Lamie: conceptualization, data curation, investigation, methodology, formal analysis, and writing original draft. Enas Elmowafy: conceptualization, data curation, investigation, formal analysis, validation, supervision, and writing review and editing. Maha H. Ragaei: methodology, data analysis and curation, and writing review and editing. Dalia A. Attia: conceptualization, data curation, formal analysis, supervision, and writing review and editing. Nahed D. Mortada: conceptualization, formal analysis, data curation, supervision, and writing review and editing.

## Supplementary Material

Supplemental Material
