# Supplementary material for: Assessment of antifungal efficacy of itraconazole loaded aspasomal cream: comparative clinical study
Source: Drug Deliv. 2022 May 4;29(1):1345–57. doi: 10.1080/10717544.2022.2067601 (PMC9090397; doi:10.1080/10717544.2022.2067601)
Supplement: Supplemental Material [file IDRD_A_2067601_SM2563.docx]

**Supplementary Information**

**Assessment of antifungal efficacy of Itraconazole loaded aspasomal cream: Comparative clinical study**

**Authors and affiliations**

**Caroline Lamie^1^, Enas Elmowafy^2^, Maha H. Ragaie^3^, Dalia A. Attia ^1^, Nahed D. Mortada^2^**

**^1^ Pharmaceutics and Pharmaceutical Technology, The British University in Egypt.**

**^2^ Pharmaceutics and Industrial Pharmacy Department, Faculty of Pharmacy, Ain**

**Shams University, Cairo, Egypt.**

**^3^ Department of Dermatology, STD’s and Andrology, Faculty of Medicine, Minia University, Al Minya, Egypt.**

**Corresponding author**

**Dalia A. Attia**

Department of Pharmaceutics and Pharmaceutical Technology, The British University in Egypt (BUE), Cairo, Egypt

Tel.:(+202)01111414144 Fax:(+202)26300010/20

Email: dalia.rhman@bue.edu.eg

**Table S1:** The obtained evaluation criteria and values of 0.1% and 0.5% aspasomal cream

| **Evaluation criteria** | **ITZ aspasomal cream** | |
| --- | --- | --- |
|  | **0.1%^a^** | **0.5%** |
| **1-Drug content** | **95.12% ± 0.24** | **96.41% ± 1.1** |
| **2- pH** | **6.84 ±0.21** | **6.84 ±0.32** |
| **3-Spreadability** | **443.3% ± 6.5** | **442.4% ± 5.1** |
| **4- Occlusive effect** | **78.26% ±5.75** | **79.06% ±3.7** |
| **5- Extrudability** | **97.5% ± 0.82** | **96.6% ± 1.2** |
| **6- Viscosity** | **pseudoplastic (shear-thinning) behavior** | |

**^a^ 0.1% aspasomal cream formulae prepared earlier by our group (recently accepted paper)**

**Table S2:** represent the stability study of the prepared 0.1%, 0.5% aspasomal cream

| **Stability evaluation criteria** | **ITZ aspasomal cream** | |
| --- | --- | --- |
|  | **0.1%** | **0.5%** |
| **1- Drug content** | **95% ± 1.2** | **96.2% ± 0.45** |
| **2- pH** | **6.71 ±0.4** | **6.75 ±0.32** |
| **3- Spreadability** | **443.3% ± 4.5** | **442.4% ± 2.1** |
| **4- Occlusive effect** | **77.35% ± 4.25** | **78.14% ± 2.8** |
| **5- Extrudability** | **97.4%± 0.57** | **96.4% ± 0.97** |
| **6- Viscosity** | **pseudoplastic (shear-thinning) behavior** | |

**Table S3**: Demographic data

|  |  |  |  | **FC1** |  | **FC1’** |  | **FC2** |  | **FC2’** |  | **FC3** |  | **FC4** |  | ***P*-value** |
| --- | --- | --- | --- | --- | --- | --- | --- | --- | --- | --- | --- | --- | --- | --- | --- | --- |
| **Age in months^1^** |  | *Range* |  | (5-24) |  | (6-25) |  | (6-25) |  | (6-25) |  | (7-17) |  | (5-22) |  | 0.868 |
|  |  | *Mean ± SD* |  | 13.5±6.1 |  | 13.7±6.8 |  | 12.6±5.6 |  | 13.2±6.9 |  | 10.8±2.7 |  | 13.6±5.4 |  |  |
| **Sex^2^** |  | *Male* |  | 4(40%) |  | 6(60%) |  | 5(50%) |  | 6(60%) |  | 4(40%) |  | 6(60%) |  | 0.911 |
|  |  | *Female* |  | 6(60%) |  | 4(40%) |  | 5(50%) |  | 4(40%) |  | 6(60%) |  | 4(40%) |  |  |
| **Duration of disease^3^ (Days)** |  | *Median* |  | 9 |  | 8 |  | 7 |  | 10 |  | 9 |  | 12.5 |  | 0.839 |
|  |  | *IQR* |  | (7-16.5) |  | (7-10.8) |  | (6.5-16.5) |  | (7-15) |  | (7-16.5) |  | (6-21) |  |  |

- ***^1^One Way ANOVA test between the 6groups followed by post hoc analysis between each two groups.***
- ***^2^Fisher’s Exact test for qualitative data between groups.***
- ***^3^Kruskal Wallis test between the 6groups followed by Mann Whitney test between each two groups.***
- ****Significant level at P value ≤ 0.05.***

### Table S4: Demographic data

|  |  |  |  | **FC1** |  | **FC1’** |  | **FC2** |  | **FC2’** |  | **FC3** |  | **FC4** |  | ***P*-value** |
| --- | --- | --- | --- | --- | --- | --- | --- | --- | --- | --- | --- | --- | --- | --- | --- | --- |
| **Age in years^1^** |  | *Range* |  | (6-25) |  | (5-24) |  | (7-22) |  | (6-20) |  | (8-26) |  | (5-22) |  | 0.878 |
|  |  | *Mean±SD* |  | 13.4±6.2 |  | 13.3±5.9 |  | 13±5.5 |  | 14±4.4 |  | 15.7±6.1 |  | 15.8±.5 |  |  |
| **Sex^2^** |  | *Male* |  | 6(60%) |  | 4(40%) |  | 5(50%) |  | 6(60%) |  | 4(40%) |  | 5(50%) |  | 0.953 |
|  |  | *Female* |  | 4(40%) |  | 6(60%) |  | 5(50%) |  | 4(40%) |  | 6(60%) |  | 5(50%) |  |  |
| **Duration of disease^3^(Days)** |  | *Median* |  | 8.5 |  | 7 |  | 9.5 |  | 12.5 |  | 10 |  | 10.5 |  | 0.596 |
|  |  | *IQR* |  | (7-15) |  | (6.5-16.5) |  | (6.8-15) |  | (9.3-20.3) |  | (6.8-14.3) |  | (7.8-17.3) |  |  |

- ***^1^Kruskal Wallis test between the 6 groups followed by Mann Whitney test between each two groups***
- ***^2^Fisher’s Exact test for qualitative data between groups.***
- ***^3^Significant level at P value < 0.05.***





**Fig S1: Viscosity measurements of the 0.1% and 0.5% ITZ aspasomal creams. (n=3±SD)**.

**^a^ 0.1% aspasomal cream formulae prepared earlier by our group (recently accepted paper)**

**
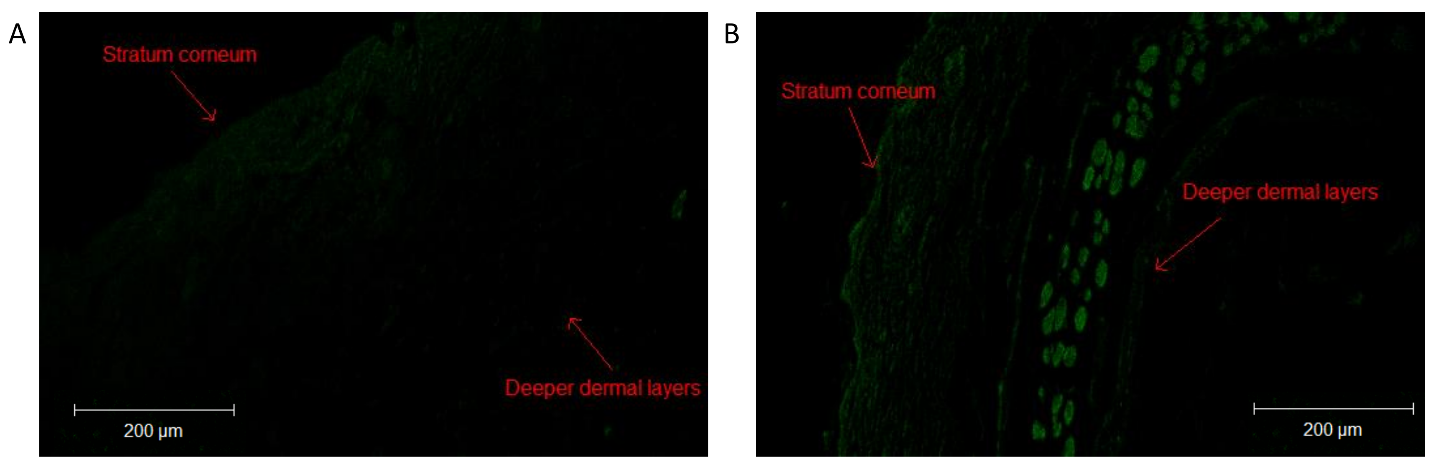
**

**Figure S2: CLSM images showing cross-sectional views of mice skin treated with (A) Dil fluorescent dye in cream, (B) aspasomal cream after 24-h application.**
